# Concrete Mix Design for Completely Recycled Fine Aggregate by Modified Packing Density Method

**DOI:** 10.3390/ma13163535

**Published:** 2020-08-11

**Authors:** Yibo Yang, Baixi Chen, Yan Su, Qianpu Chen, Zhiji Li, Wenying Guo, Hengchang Wang

**Affiliations:** 1 School of Civil Engineering and Transportation, South China University of Technology, Guangzhou 510640, China; yangyibo@scut.edu.cn (Y.Y.); suyan@mail.scut.edu.cn (Y.S.); chenqp@kaisagroup.com (Q.C.); ctzhjli@mail.scut.edu.cn (Z.L.); wyguo@scut.edu.cn (W.G.); cthcwang@scut.edu.cn (H.W.); 2 State Key Laboratory of Subtropical Building Science, South China University of Technology, Guangzhou 510640, China; 3 School of Civil Engineering, The University of Sydney, Sydney, NSW 2006, Australia

**Keywords:** concrete waste, completely recycled fine aggregate, concrete mix design, modified packing density method

## Abstract

The undesirable properties of conventional recycled fine aggregate (RFA) often limit its application in the construction industry. To overcome this challenge, a method for preparing completely recycled fine aggregate (CRFA), which crushes all concrete waste only into fine aggregate, was proposed. The obtained CRFA had high apparent density, and its water absorption was lower than that of the conventional RFA. To take advantage of the CRFA, this paper introduced the modified packing density method for the CRFA concrete mix design. The modified packing density method took account of the powder with a particle size of smaller than 75 μm in the CRFA and balanced both the void ratio and the specific surface area of the aggregate system. Concrete (grade C55) was prepared using the CRFA to validate the feasibility of the proposed method. The unit price of the prepared CRFA concrete was around 12.7% lower than that of the natural aggregate concrete. Additionally, the proposed procedure for the concrete mixture design could recycle all concrete waste into the new concrete and replace all the natural fine aggregate in the concrete mixture.

## 1. Introduction

With plenty of urbanization happening over the world, the construction industry gradually becomes the major consumer of the natural resources in the world. Thus, many developing countries face a shortage of natural fine aggregate and the difficulty of dealing with concrete waste. According to the statistics, the global aggregate production has increased from 21 to 40 billion tons from 2007 to 2014 [1]. On the other hand, a huge volume of construction and demolition waste (CDW) is produced from fast urbanization. Department of Environmental Affairs [2] stated that 43.78 million tons of CDW were generated annually in Australia during 2006–2007, and China’s annual production of CDW reaching 180 million tons in 2018 has been increasing without stop [3]. CDW is difficult to be degraded and needs to be buried underground generally [4], which not only occupies the land but also pollutes the environment. Ordinarily, concrete waste accounts for 30 to 40% of total CDW [5], so separating concrete waste and crushing it into recycled aggregate can provide an effective way to solve both problems.

Since concrete is made of natural coarse aggregate, natural fine aggregate, and cementitious material, recycled concrete waste is usually classified as recycled aggregate (RA), i.e., recycled coarse aggregate (RCA) or recycled fine aggregate (RFA), and recycled powder. Recycled aggregate consists of original aggregate and the adhered mortar. The porosity of the adhered mortar distinguishes recycled aggregate from the natural aggregate and causes the recycled aggregate to have lower density and higher water absorption. Hence, the quality of recycled aggregate mostly depends on the quality and amount of the adhered mortar [6].

In the conventional recycling procedure, larger recycled aggregate particles are separated from the crushed concrete and used as RCA, and the smaller ones are used as RFA. For this procedure, some researches [7,8,9] have stated that the quantity of the adhered mortar rises when the average particle size of the recycled aggregate decreases, which makes the water absorption capacity of RFA higher than that of RCA. Therefore, RCA, which presents mechanical properties similar to the natural aggregate [10,11,12], is the main application of concrete waste, and RFA is often avoided to be used. Nevertheless, the shortage of natural fine aggregate has recently drawn the attention of researchers and led to many studies on RFA. For instance, Kou and Poon [13] stated that using RFA could decrease the compressive strength of concrete but could increase concrete drying shrinkage. Some studies [14,15] have illustrated that the addition of RFA at percentages lower than 30% does not lower the mechanical properties and durability of concrete. When the percentage of RFA is over 50%, however, the workability of the fresh concrete paste is significantly affected [16]. In order to avoid the adverse effect, Chinese Code JGJ/T240-2011 "Technical Specification for Application of Recycled Aggregate” [17] points out that the replacement ratio of RFA should not be greater than 50%, and it should not be used in the concrete whose strength grade is higher than C40. It should be noted that the strength grade of concrete is expressed by “C” + “the characteristic value of cubic concrete compressive strength” here.

In order to further enhance the properties of RFA and increase the utilization rate of concrete waste, Yang et al. [18] introduced a procedure, which crushes concrete waste into RFA completely without generating RCA. This procedure is called completely recycled fine aggregate (CRFA) technology and has been granted the Chinese patent.

In the conventional procedure, a natural coarse aggregate that accounts for 40% of concrete waste is often sieved into RCA instead of RFA, which raises the percentage of mortar in RFA. In contrast, CRFA could make use of this part of natural coarse aggregate since it crushes all concrete waste into fine aggregate. Regarding the crushed products of natural coarse aggregate, the ratio of mortar in CRFA is lower than that in conventional RFA, and the apparent density and porosity of CRFA are better than those of conventional RFA [19]. The advantages of CRFA improve its usability greatly and make it possible for CRFA to replace all natural fine aggregate used in concrete. Fan et al. [20,21] reached a similar conclusion as well and stated that crushing all concrete waste into RFA could produce RFA with a smoother surface texture. However, different from the CRFA preparation method, the procedure reported by Fan et al. further separated most of the powder (particles smaller than 150 μm) from the obtained RFA by a roller sand washer. Considering the high cost of separating the powder with a particle size of smaller than 150 μm from the obtained RFA, CRFA retains and exploits this part as the fine aggregate as well.

For CRFA concrete mix design, the packing density method should be an acceptable approach. It minimizes the void ratio of the aggregate system and the consumption of the cement paste by maximizing the bulk density of the blended aggregate [22,23]. The ratio of fine aggregate given by the packing density method is high, which may be suitable for natural aggregate, but not good for CRFA. As the powder content of CRFA is higher than that of natural aggregate, the high fine aggregate ratio expands the specific surface area of CRFA, thereby reducing the concrete workability.

Since there is not a suitable mix design method available for the CRFA concrete currently, it is difficult to apply the CRFA in the construction industry. In order to solve this problem, this work proposed a modified packing density method, which can not only take the feature of the CRFA into account but also provide a balance between the low void ratio and the low specific surface area. In the rest of the paper, the preparation process of the CRFA and the modified packing density method for the CRFA concrete mix design are introduced in Section 2. The properties of the CRFA are discussed in Section 3.1, and the preparation of the CRFA concrete (grade C55) used to validate the mix design procedure is described in Section 3.2; finally, Section 4 summarizes the results obtained.

## 2. Materials and Methods

### 2.1. Preparation Process of the CRFA

Due to the size limitation of the pilot plant, the steps to remove the impurities of concrete waste and crush them into concrete blocks smaller than 200 mm are not included in the preparation process. The pilot plant preparation process of the CRFA can be divided into two main stages.

In the first stage, the concrete waste is crushed by PE250 × 400 jaw crusher (Guangdong Leimeng Heavy Machinery Manufacturing Co., Ltd., Guangzhou, China). The size of the obtained aggregate can be controlled by adjusting the outlet size of the jaw crusher. All the concrete waste blocks smaller than 200 mm are crushed into blocks smaller than 50 mm.

**Figure 1:**
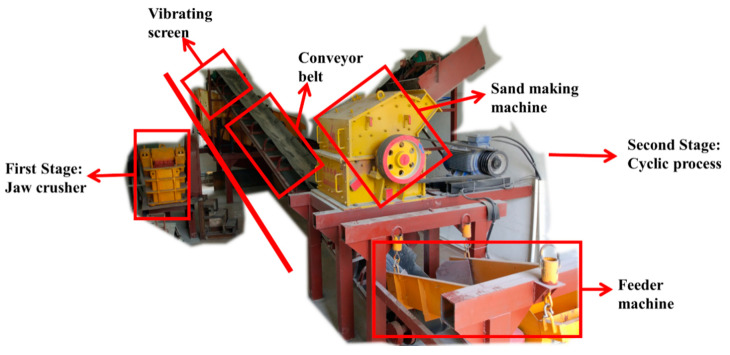
The equipment for the production of the completely recycled fine aggregate (CRFA) on a pilot scale.

The second stage is the cyclic sand making. The blocks smaller than 50 mm are crushed repeatedly until all the aggregate has an average particle size of less than 4.75 mm. The obtained aggregate with an average particle size of smaller than 4.75 mm is called the CRFA. The equipment used in this cyclic process is composed of a feeder machine, a conveyor belt, a vibrating screen, and the PCX8040 sand making machine (hammer crusher, Guangdong Leimeng Heavy Machinery Manufacturing Co., Ltd., Guangzhou, China). During the procedure, the whole powder produced is retained in the CRFA. The equipment is displayed in Figure 1.

### 2.2. Modified Packing Density Method for the CRFA Concrete Mix Design

When the conventional packing density method is applied to the concrete mix design, it designs the aggregate mixture with the highest density, i.e., maximum packing [24]. Although this kind of approach can reduce the amount of binder and lower the shrinkage and creep of the concrete, the mass fraction of the fine aggregate (MFF) in the optimal aggregate mixture is high [22]. As there is much powder in the CRFA, a high MFF may lead to the large specific surface area of the aggregate and worsen the concrete workability. Thus, to suit the packing density method to the CRFA, the modified packing density method is proposed in this section. Instead of choosing an MFF, which leads to the largest packing density like the conventional approach, the modified packing density method uses the MFF above which the increment in the packing density is not considerable. At the MFF given by the modified approach, both the void content and the specific surface area of the aggregate mixture can be maintained on a low level. Since there is not much powder in the coarse aggregate, the proportion of the coarse aggregate can still be determined by the conventional packing density method. As the packing density correlates positively with the bulk density [25], the bulk density is measured for the proportion selection in place of the packing density from now on.

After the proportion of the aggregate mixture is obtained, the water-to-cement (W/C) ratio can be determined based on the strength requirement. The volume of the cement paste is equal to the void content of the combined aggregate. In order to coat the aggregate particles and obtain a workable concrete mixture, the paste content should be higher than the void content [22]. The ratio of the paste content to the void content is called the excess factor (EF), and the recommended range of EF is 1.05–1.15 [22]. The amount of water and cement can be calculated based on the volume of the cement paste and the W/C ratio.

As the water absorption of the CRFA is higher than that of natural aggregate, additional water is required to improve the workability of the CRFA concrete. However, adding too much water will reduce the concrete strength since the CRFA cannot retain the additional water strongly, and part of the absorbed water may flow into the concrete mixture and influence the W/C ratio. In order to take account of this effect, the saturation factor (SF) is introduced herein to evaluate the degree of the saturation of the CRFA; the recommended range of SF is 0–60% [19]. The CRFA concrete can achieve the same mechanical performance as the natural aggregate concrete by adjusting the SF. Moreover, water reducer can be added to improve the behavior of CRFA concrete.

For further reducing the cement content and the MFF of the CRFA concrete, a three-stage optimization is conducted. In the first stage, the EF is optimized, and the minimum value satisfying the workability requirement is regarded as the optimal EF. In the second stage, the cement paste is replaced with the CRFA of the same volume to further lower the paste content. The ratio between the paste content after and before the replacement is called the cement paste index (CPI). The slump test is conducted to determine the minimum CPI for workable concrete mixtures. In the third stage, the CRFA is replaced by a natural coarse aggregate of the same volume for decreasing the specific surface area of the aggregate mixture. The minimum MFF satisfying the concrete workability requirement is regarded as the optimal MFF. After the three-stage optimization, the obtained concrete mixture proportion has a low cement paste content, and the obtained aggregate mixture has both a low void content and a low specific surface area.

**Figure 2:**
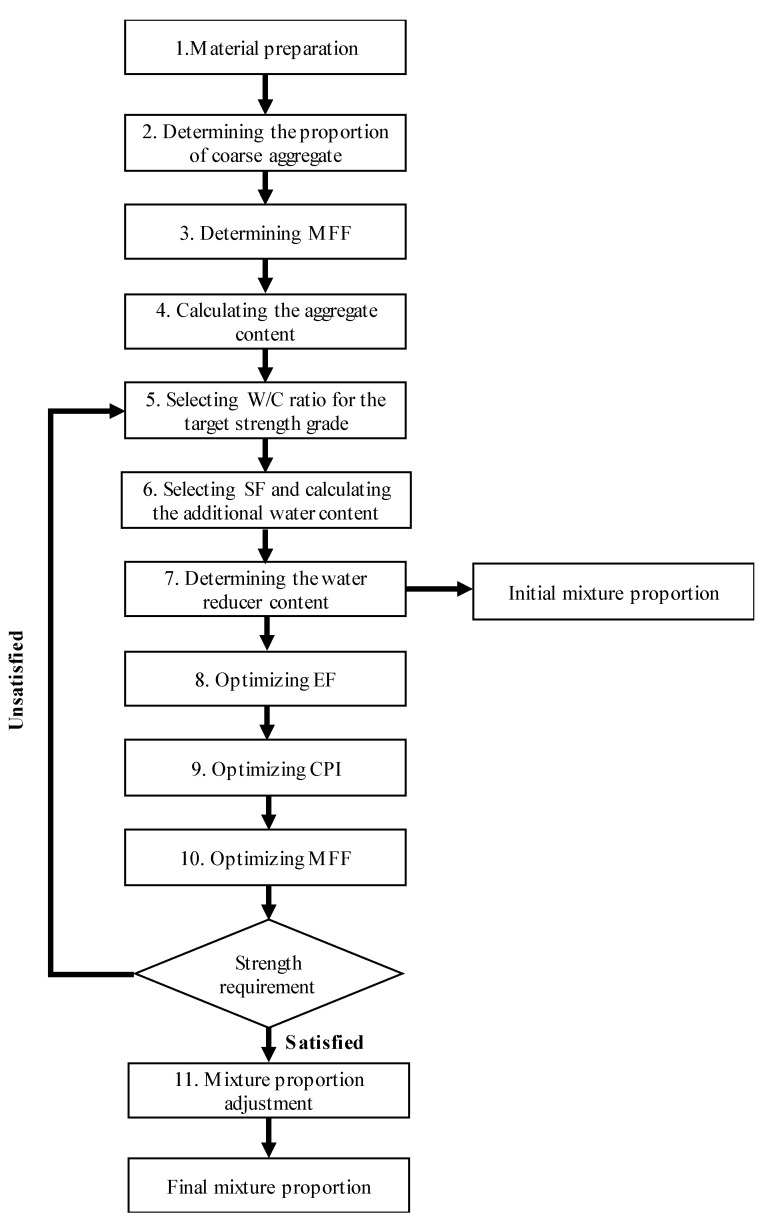
Flow chart of the procedure proposed for concrete mix design.

The procedure for the proposed CRFA concrete mixture design is outlined in Figure 2, and the step-by-step procedure is summarized below:

**Step 1.**  Preparing the materials

The CRFA and coarse aggregate are dried, and their properties (density, water absorption, etc.) are measured.

**Step 2.**  Determining the proportion of coarse aggregate of different sizes

The proportion that maximizes the compacted bulk density of the coarse aggregate mixture is selected as the proportion of the coarse aggregate.

**Step 3.**  Determining MFF

The CRFA is added into the coarse aggregate mixture obtained in the previous step. Then, the MFF above which the increment in the bulk density is not remarkable is selected as the initial MFF.

**Step 4.**  Calculating the aggregate content

The aggregate content for 1 m^3^ of concrete can be calculated by Equations (1) and (2):

(1) $$m_{f}=\rho_{b}\times \mathrm{MFF}$$

(2) $$m_{ci}=\rho_{b}\times {\mathrm{MFC}}_{i}$$

$m_{f}$: the mass of the CRFA in 1 m^3^ of concrete (kg);
$m_{ci}$: the mass of coarse aggregate *i* in 1 m^3^ of concrete (kg);
$\rho_{b}$: the bulk density corresponding to the MFF obtained in step 3 (kg/m^3^);
MFF: the mass fraction of the fine aggregate;
${\mathrm{MFC}}_{i}$: the mass fraction of coarse aggregate *i*.

**Step 5.**  Selecting the W/C ratio for the target strength grade

The W/C ratio of the CRFA concrete is the same as that of the natural aggregate concrete. The content of the water and the cement can be calculated by Equations (3) and (4), respectively:

(3) $$m_{c}=\frac{\rho_{c}\times \rho_{w}\times EF\times V_{V}}{\rho_{w}+W/C\;\mathrm{ratio}\times \rho_{c}}$$

(4) $$m_{wc}=W/C\;\mathrm{ratio}\times m_{c}$$

(5) $$V_{V}=1-\frac{m_{f}}{\rho_{f}}-\sum \frac{m_{ci}}{\rho_{ci}}$$

$m_{c}$: the mass of cement in 1 m^3^ of concrete (kg);
$m_{wc}$: the mass of the water included in W/C ratio in 1 m^3^ of concrete (kg);
$\rho_{c}$: the density of the cement (kg/m^3^);
$\rho_{w}$: the density of the water (kg/m^3^);
$\rho_{f}$: the apparent density of the CRFA (kg/m^3^);
$\rho_{ci}$: the apparent density of coarse aggregate *i* (kg/m^3^);
$V_{V}$: the void volume (m^3^);
EF: the excess factor; the initial value is 1.05.

**Step 6.**  Selecting SF and calculating the additional water content

An SF is selected based on the W/C ratio. The large SF values are used for the concrete with a low W/C ratio, and the small values are used for the concrete with a high W/C ratio. For example, an SF of 10% is used for the W/C ratio of 0.50, and an SF of 60% is used for the W/C ratio of 0.33. The content of the additional water can be calculated by Equation (6):

(6) $$m_{wa}=m_{f}\times \beta \times SF$$

$m_{wa}$: the mass of the additional water in 1 m^3^ of concrete (kg);
β: the saturated-surface-dry water absorption of the CRFA.

**Step 7.**  Determining the water reducer content

The water reducer content ($m_{wr}$) that offers the best workability of the concrete is selected. The initial mixture proportion can also be obtained in this step, and the total water content of the initial mixture proportion can be defined as:

(7) $$m_{w}=m_{wc}+m_{wa}-m_{ww}$$

$m_{w}$: the mass of the water in 1 m^3^ of concrete (kg);
$m_{ww}$: the mass of the water in the water reducer (kg). For the liquid water reducer, it is approximately equal to the mass of the water reducer, i.e., $m_{wr}$.

In the optimization stage (steps 8–10), the content of the water reducer can be adjusted to achieve the best fresh performance of the concrete.

**Step 8.**  Optimizing EF

The workability test is conducted on the concrete with different excess factors, and the minimum value satisfying the workability requirement is regarded as the optimal EF. For different excess factors, the amount of water and cement needs to be recalculated by Equations (3), (4), and (7).

**Step 9.**  Optimizing CPI

The workability test is conducted on the concrete with different cement paste indices, and the minimum value satisfying the workability requirement is regarded as the optimal CPI. For different cement paste indices, the content of the water, the CRFA, and the cement need to be recalculated by Equation (4) and Equations (6)–(9). $m_{c}$ and $m_{f}$ in Equation (4) and Equation (6) are replaced by $m_{c}^{\prime }$ and $m_{f}^{\prime }$, respectively.

(8) $$m_{f}^{\prime }=m_{f}+\left(1-CPI\right)\times EF\times V_{V}\times \rho_{f}$$

(9) $$m_{c}^{\prime }=\frac{\rho_{c}\times \rho_{w}\times CPI\times EF\times V_{V}}{\rho_{w}+W/C\;\mathrm{ratio}\times \rho_{c}}$$

$m_{f}^{\prime }$: the recalculated mass of the CRFA in 1 m^3^ of concrete in step 9 (kg);
$m_{c}^{\prime }$: the recalculated mass of cement in 1 m^3^ of concrete in step 9 (kg).

**Step 10.**  Optimizing MFF

The workability test is conducted on the concrete with different mass fractions of the fine aggregate, and the minimum value satisfying the concrete workability requirement is regarded as the optimal MFF. For different MFFs, the content of the water, the CRFA, and the coarse aggregate need to be recalculated by Equations (6), (7), (10), and (11). $m_{f}$ in Equation (6) is replaced by $m_{f}^{\prime \prime }$.

(10) $$m_{f}^{\prime \prime }=m_{f}^{\prime }-∆m_{f}$$

(11) $$m_{ci}^{\prime \prime }=m_{ci}+∆m_{f}\times \frac{{\mathrm{MFC}}_{i}}{\sum {\mathrm{MFC}}_{i}}\times \frac{\rho_{ci}}{\rho_{f}}$$

(12) $$∆m_{f}=\frac{m_{f}^{\prime }-\mathrm{MFF}\times \left(m_{f}^{\prime }+\sum m_{ci}\right)}{\mathrm{MFF}\times \left(\sum \left(\frac{{\mathrm{MFC}}_{i}}{\sum {\mathrm{MFC}}_{i}}\times \frac{\rho_{ci}}{\rho_{f}}\right)-1\right)+1}$$

$m_{f}^{\prime \prime }$: the recalculated mass of the CRFA in 1 m^3^ of concrete in step 10 (kg);
$m_{ci}^{\prime \prime }$: the recalculated mass of coarse aggregate *i* in 1 m^3^ of concrete in step 10 (kg);
$∆m_{f}$”: the change in the mass of the CRFA for different MFFs (kg).

The obtained optimal proportion is checked for meeting the strength requirement. If the strength requirement is not satisfied, we go back to step 5 and reselect the W/C ratio and SF.

**Step 11.**  Mixture proportion adjustment

The concrete mixture proportion is adjusted by the concrete apparent density. The adjusted material content of 1 m^3^ of concrete is calculated by:

(13) $$m_{f}^{*}=m_{f}^{\prime \prime }\times \frac{\rho_{concrete}}{m_{f}^{\prime \prime }+\sum m_{ci}^{\prime \prime }+m_{w}+m_{c}^{\prime }+m_{wr}}$$

(14) $$m_{ci}^{*}=m_{ci}^{\prime \prime }\times \frac{\rho_{concrete}}{m_{f}^{\prime \prime }+\sum m_{ci}^{\prime \prime }+m_{w}+m_{c}^{\prime }+m_{wr}}$$

(15) $$m_{w}^{*}=m_{w}\times \frac{\rho_{concrete}}{m_{f}^{\prime \prime }+\sum m_{ci}^{\prime \prime }+m_{w}+m_{c}^{\prime }+m_{wr}}$$

(16) $$m_{c}^{*}=m_{c}^{\prime }\times \frac{\rho_{concrete}}{m_{f}^{\prime \prime }+\sum m_{ci}^{\prime \prime }+m_{w}+m_{c}^{\prime }+m_{wr}}$$

(17) $$m_{wr}^{*}=m_{wr}\times \frac{\rho_{concrete}}{m_{f}^{\prime \prime }+\sum m_{ci}^{\prime \prime }+m_{w}+m_{c}^{\prime }+m_{wr}}$$

$m_{f}^{*}$: the mass of the CRFA in 1 m^3^ of concrete at the optimal proportion (kg);
$m_{ci}^{*}$: the mass of coarse aggregate *i* in 1 m^3^ of concrete at the optimal proportion (kg);
$m_{w}^{*}$: the mass of water in 1 m^3^ of concrete at the optimal proportion (kg);
$m_{c}^{*}$: the mass of cement in 1 m^3^ of concrete at the optimal proportion (kg);
$m_{wr}^{*}$: the mass of water reducer in 1 m^3^ of concrete at the optimal proportion (kg);
$\rho_{concrete}$: the concrete apparent density (kg/m^3^).

### 2.3. Materials

#### 2.3.1. Cement

The properties of P.II 42.5R Portland cement used in this work are listed in Table 1.

#### 2.3.2. Concrete Waste

The concrete waste (strength grade: C40) used for preparing the CRFA was obtained from the blocks of the hollow slabs and beams of a highway viaduct. The coarse aggregate and the fine aggregate of the concrete waste were the crushed granite stone and the river sand, respectively.

#### 2.3.3. Coarse Aggregate

Two types of natural coarse aggregate (crushed stone) with a particle size of 10 and 20 mm were used. The properties of the crushed stones are presented in Table 2.

#### 2.3.4. Admixture

HG7 polycarboxylate water reducer at a solid content of 20% was employed as the admixture.

### 2.4. CRFA Testing Method

Eight physical properties of the CRFA, namely, particle gradation, fineness module, the content of fine powder, crush index, apparent density, saturated-surface-dry water absorption, loose bulk density, and loose void volume, were tested by the methods suggested in standard GB/T 25176-2010 “Recycled Fine Aggregate for Concrete and Mortar” [26] and standard GB14684-2011 “Sand for Construction” [27].

The particle appearance of the CRFA of five different particle sizes of 1.18–2.36 mm, 0.60–1.18 mm, 0.30–0.60 mm, 0.15–0.30 mm, and 0–0.15 mm was also analyzed using Olympus, SZX10 stereomicroscope (Olympus Corporation, Tokyo, Japan). The powder particles smaller than 0.15 mm were sieved by a 0.080 mm standard cement negative pressure screen. After that, the dried powder particles smaller than 0.080 mm were divided into two samples. One sample was gilded by Cressington 108 Sputter Coater (Cressington Scientific Instruments Ltd., Watford, England (UK)), and microcosmic analysis was conducted on it by ZEISS EVO-18 Premium scanning electron microscope (Carl Zeiss AG, Oberkochen, Germany). The particle distribution of the other sample was measured by using the Horiba LA-950 laser particle size analyzer (Horiba Ltd., Kyoto, Japan).

### 2.5. Concrete Sample Design and Testing Method

For each concrete mixture proportion, the initial slump, the slump after 1 h, the initial slump-flow, and the slump-flow after 1 h were tested by the methods recommended in GB/T 50080-2016, “Standard for The Test Method of Performance on Ordinary Fresh Concrete” [28]. Cubic samples with the dimensions 100 mm × 100 mm × 100 mm were cast and put indoor for 24 h before demolding. Afterward, the samples were cured in a standard curing room at a temperature of 20 °C and relative humidity of 95%. The compressive strength of the samples after 3, 7, and 28 days of curing was tested by the methods described in standard GB/T 50081-2002 [29].

## 3. Results and Discussion

### 3.1. Performance of CRFA

**Figure 3:**
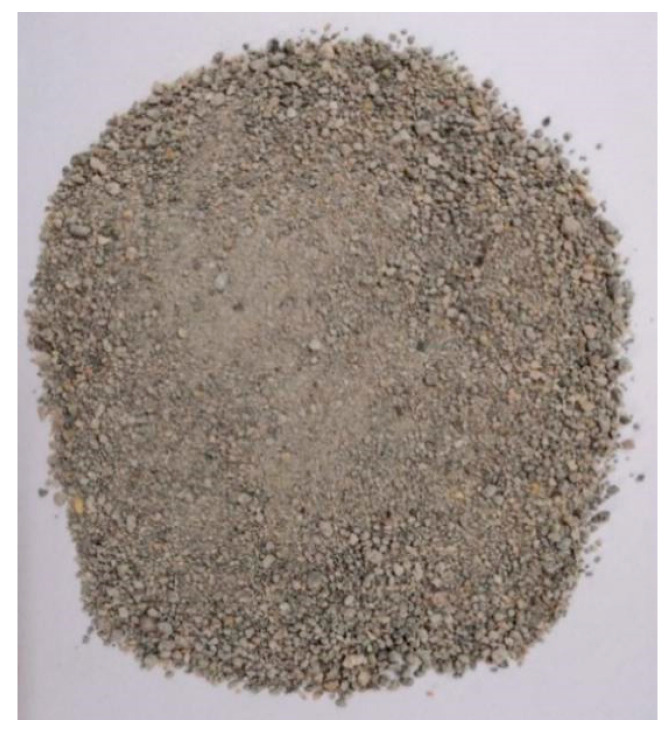
The appearance of the produced CRFA.

**Figure 4:**
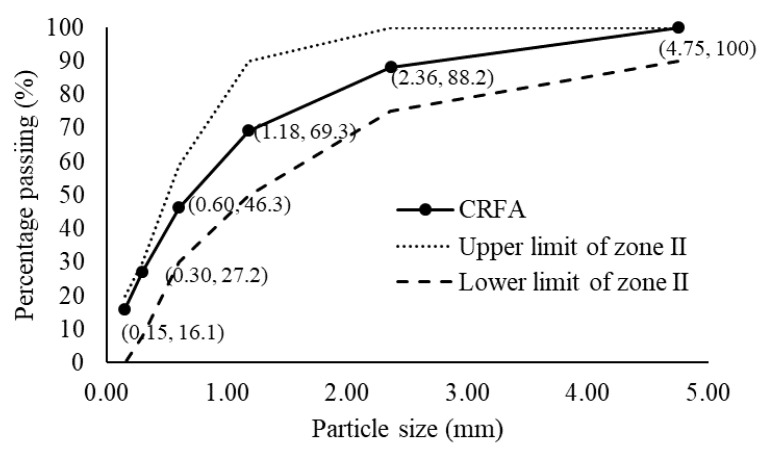
The particle grading curve of the produced CRFA.

The appearance and particle grading of the CRFA produced by the procedure described in Section 2.1 are illustrated in Figure 3 and Figure 4, respectively, and its physical properties are listed in Table 3. According to code GB/T 25176-2010 [26], the CRFA is considered to be medium sand, and the particle grading is suitable and belongs to zone II; moreover, the apparent density, the bulk density, and the porosity have reached the requirement of the first-class sand; the crush index also meets the requirement of the second class sand. However, since the fine powder in the CRFA is not sieved, the content of the fine powder is high, but it satisfies the third class sand requirement. The water absorption of the saturated-surface-dry CRFA (6.4%) is smaller than that of the conventional recycled aggregate, averaging 9.9% with a standard deviation of 2.4% [30]. Overall, the quality of the produced CRFA is high.

**Figure 5:**
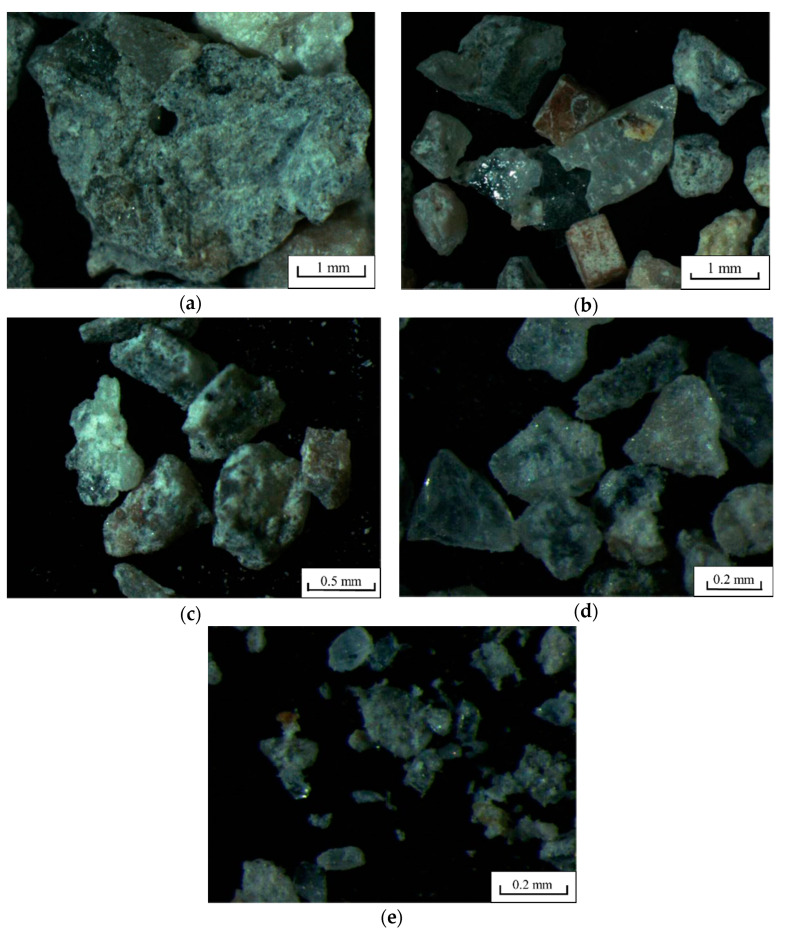
The appearance of the CRFA with different particle sizes under the stereomicroscope. (**a**) 1.18–2.36 mm, (**b**) 0.60–1.18 mm, (**c**) 0.30–0.60 mm, (**d**) 0.15–0.30 mm, (**e**) 0–0.15 mm.

Figure 5 illustrates the particle appearance of the CRFA with different particle sizes under the stereomicroscope. It can be observed that the crushed stones adhered by the mortar are the main component of the CRFA, so the CRFA meets the conditions of including 40% crushed products of natural coarse aggregate. It can also be seen in Figure 5a that there are plenty of pores in the mortar, which leads to the higher water absorption and lower apparent density of the CRFA compared to river sands. The CRFA has an irregular pointed shape that is generated during crushing; the sharp edges and corners can be removed through the collision of the aggregate particles with the hammer of the sand making machine and with other particles. However, the aggregate is sieved out when it is crushed into the particles smaller than 4.75 mm, so the sieved aggregate lacks sufficient collisions and still has many edges and corners.

**Figure 6:**
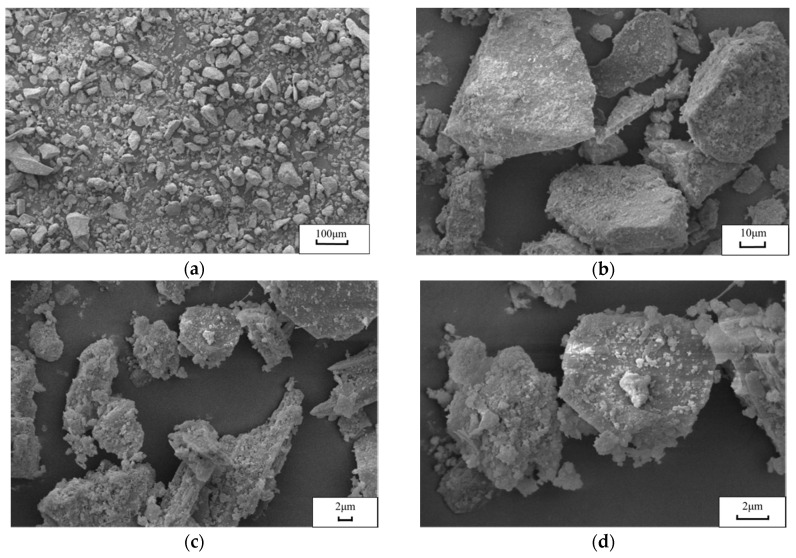
The scanning electron microscope images of the CRFA powder. (**a**) 100×, (**b**) 800×, (**c**) 2000×, (**d**) 5000×.

Figure 6 displays the scanning electron microscope (SEM) images of the CRFA powder with a particle size of less than 0.080 mm. It can be seen in Figure 6a, at a magnification of 100×, and Figure 6b, at a magnification of 800×, that most of the powder is composed of stones and mortar, and the powder particles have an irregular shape and undesirable sphericity. According to Figure 6c,d, presenting SEM images at a magnification of 2000× and 5000×, respectively, the powder is highly porous, which is the main reason for its high water absorption. Since the powder with a particle size of smaller than 0.080 mm and the one with a particle size between 0.080 and 4.75 mm are highly porous, the obtained CRFA has higher water absorption compared to natural fine aggregate.

**Figure 7:**
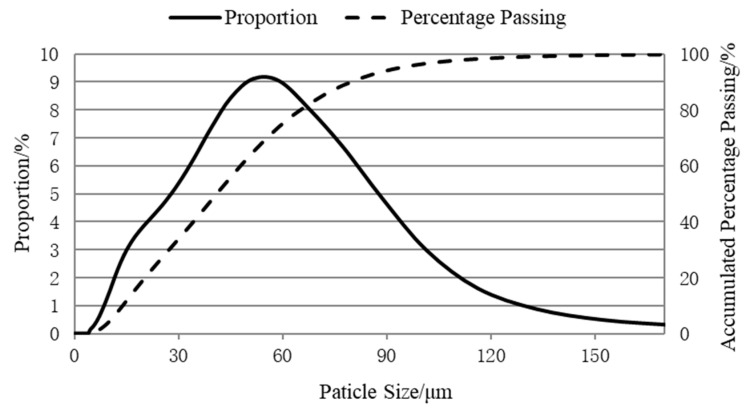
The particle size distribution of the CRFA powder with a particle size of smaller than 0.080 mm.

The CRFA powder with a particle size of smaller than 0.080 mm was characterized using laser particle size analysis. The particle size distribution of the powder is presented in Figure 7, and Table 4 tabulates its characteristic particle sizes. The average particle size of the CRFA powder is 44.9 μm, and 90% of the powder particles are smaller than 80.2 μm. It can be seen that 10% of the powder particles are larger than 0.080 mm, which is because the sieve method uses the diameter of the inscribed sphere of the particle, but the laser particle size analyzer measures the particle size in one direction. When the powder has undesirable sphericity, the particle size calculated by the laser particle size analyzer is larger than that measured by the sieve method, so the results of the two methods are not in agreement [31]. Fan and Wang [32] stated that the average particle size of cement is around 22 μm; thus, since the particle size of the CRFA powder is close to that of cement, we may substitute the CRFA powder for the cement to fill up the void of the aggregate system.

### 3.2. CRFA Concrete (Grade C55) Design

The mixture of the CRFA concrete (grade C55) was designed by following the procedure described in Section 2.2.

All the aggregate was dried according to step 1. In step 2, two types of a coarse aggregate of different sizes, namely, crushed aggregate 1 and crushed aggregate 2, were mixed at different proportions by mass. Six different mass fractions of crushed aggregate 1, i.e., 0, 10, 15, 20, 25, and 30%, were tested here. The compacted bulk density of the coarse aggregate with six different proportions is listed in Table 5. When crushed aggregate 1 constitutes 20% of the total coarse aggregate, the maximum compacted bulk density of the coarse aggregate mixture (1532 kg/m^3^) is obtained. Thus, the optimal proportion of the coarse aggregate is 20% of crushed aggregate 1 plus 80% of crushed aggregate 2.

Following step 3, the MFF was set at 0, 36, 38, 40, 42, 44, 46, 48, and 50%. The compacted bulk density of the aggregate mixture obtained from different mass fractions of the fine aggregate is presented in Table 6. By increasing the MFF, the density of the aggregate first increases and then plateaus. After the MFF reaches 40%, the increment in the compacted bulk density is not considerable, and it vibrates around 1850 kg/m^3^; therefore, the initial MFF is 40%. The primary mix proportion of aggregate is 40% of the CRFA, 12% of crushed aggregate 1, and 48% of crushed aggregate 2. Mixed at this proportion, the aggregate mixture has both a low void content and a low specific surface area. As the compacted bulk density of 1 m^3^ of the aggregate mixture at this proportion is 1835 kg/m^3^, the content of the CRFA, crushed aggregate 1, and crushed aggregate 2 in 1 m^3^ of the concrete is equal to 734, 220, and 881 kg/m^3^, respectively.

A W/C ratio of 0.333 and an SF of 60% were selected in step 5 and step 6. The initial content of the water reducer in step 7 was set at 7.4 kg/m^3^, as determined by some pre-experiments. In the trial test, an excess factor of 1.05 just met the workability requirement, so step 8 is not reported herein. The initially calculated mixture proportion obtained from steps 1–8 is presented in Table 7. In step 9, the CPI was varied from 0.90 to 1.00, with increments of 0.05. The content of the water reducer was adjusted to achieve the best fresh performance of the concrete. The calculated mixture proportions of the concrete samples in step 9 are listed in Table 7, and their corresponding performance is presented in Table 8. When CPI decreases, the concrete workability first rises but then drops, and the best performance of the concrete is achieved at a CPI of 0.95. This phenomenon demonstrates that, by appropriately decreasing CPI, the powder of the CRFA can sufficiently be used to replace part of the cement paste when designing high-strength concrete. Hence, a CPI of 0.95 is selected, which corresponds to proportion R21.

In step 10, the CRFA is replaced with the coarse aggregate of the same volume. The mass fraction of the CRFA varies from 41.2 to 37.2%, with increments of 2%. The calculated mixture proportions and the corresponding performance of the concrete samples in step 10 are presented in Table 7 and Table 8, respectively. A reduction in the MFF has a little influence on the initial workability. However, when the MFF falls from 39.2 to 37.2%, the loss of the slump after 1 h increases significantly from 20 to 60 mm, and the loss of the slump flow rises from 10 to 160 mm. For controlling the loss of the concrete workability, an MFF of 39.2% is selected as the optimal value, and the corresponding proportion, i.e., R22, is chosen as the final mixture proportion.

Compared with the initial proportion, i.e., R11, the concrete mixed in proportion R22 has better workability and higher compressive strength. The 28-day compressive strength of the concrete mixed in proportion R22 is 64.0 MPa, which is larger than 55 + 1.654 × 5 MPa and satisfies the requirement of concrete grade C55. Furthermore, the final optimum MFF of 39.2% is smaller than the MFF of 44% measured by the conventional dry packing density method. This phenomenon resembles the wet packing density method; in fact, the MFF calculated by the wet density method is around 5% smaller than the one measured by the dry density method [25]. Therefore, the concrete mix design obtained from the modified packing density method can produce a similar effect of the wet packing density method and is more realistic.

The amount of concrete obtained by using proportion R22 shown above is often larger than 1 m^3^, so proportion R22 should be modified by using the apparent density to measure the material consumption for 1 m^3^ of the CRFA concrete. The apparent density of the concrete mixed in proportion R22 is 2304 kg/m^3^, and the final mix proportion for 1 m^3^ of the CRFA concrete is presented in Table 9.

The cost of 1 m^3^ of the CRFA concrete can be evaluated by the material prices in Guangzhou, China, as presented in Table 10. The price of the CRFA given here is the comprehensive price considering labors, energy consumptions, equipment depreciation, profits, etc. Based on the proportion in Table 9 and the material prices in Table 10, the unit price of the CRFA concrete is equal to 438.81 RMB Yuan/m^3^. The price of the natural fine aggregate concrete can be evaluated approximately by using the river sand of the same mass in the proportion; thus, the unit price of the river sand concrete is calculated as 502.78 RMB Yuan/m^3^. It can be concluded that using the CRFA is economically feasible and can reduce the concrete unit price by around 12.7%, which is attractive in the construction industry.

## 4. Conclusions

In order to improve the properties of conventional recycled fine aggregate, the CRFA is introduced in this work. Instead of crushing part of concrete waste into recycled coarse aggregate, the technique used for producing the CRFA crushes all concrete waste completely into fine aggregate. Since the crushed natural coarse aggregate in concrete waste constitutes about 40% of the CRFA, the proportion of the mortar is lower in the CRFA than in conventional recycled fine aggregate, which increases the apparent density of the CRFA and decreases the water absorption of the saturated-surface-dry CRFA.

For taking advantage of the CRFA, a procedure for the concrete mix design based on the modified dry packing density method is proposed. Different from the conventional packing density method, the modified approach chooses the mass fraction of the fine aggregate above which the increment in the packing density is not noticeable. The modified approach can take account of the fine powder in the CRFA and provide a balance between the low void content and the low specific surface area. Additionally, the modified approach can also be suitable for other fine aggregates with a high powder content.

To validate the proposed procedure for the CRFA concrete mixture, concrete grade C55 was prepared. The obtained CRFA concrete can reach the strength requirement of concrete grade C55 and offers suitable fresh performance and lower cement content. The optimum mass fraction of the fine aggregate calculated herein is 4.8% smaller than the MFF figured out by the conventional packing density method. Moreover, the CRFA concrete is well economical, and its unit price is around 12.7% lower than the natural aggregate concrete. Furthermore, by applying the proposed procedure, all concrete waste can be reused in the new concrete, and natural fine aggregate can be 100% replaced by the CRFA.

## Figures and Tables

**Table 1:** The physical and mechanical properties of the cement.

Specific Area (m^2^/kg)	Density (g/cm^3^)	Setting Time (min)		Compressive Strength (MPa)		Flexural Strength (MPa)	
		Initial	Final	3 days	28 days	3 days	28 days
362	3.14	111	165	36.4	60.0	6.4	8.9

**Table 2:** The properties of the crushed stones.

Type	Particle Size (mm)	Apparent Density (kg/m^3^)	Cumulative Sieve Residue (%)				
			19.00 mm	16.00 mm	9.50 mm	4.75 mm	2.36 mm
Crushed aggregate 1	5–10	2650	0	0	1.3	87.2	99.0
Crushed aggregate 2	5–20	2660	5.4	16.3	72.6	98.2	99.5

**Table 3:** The physical properties of the produced CRFA.

Content of fine powder (%)	9.8	Crush index (%)	21
Apparent density (kg/m^3^)	2470	Saturated-surface-dry water absorption (%)	6.4
Bulk density (loose condition) (kg/m^3^)	1350	Void content (loose condition) (%)	45
Grading zone	II	Fineness modulus	2.2

**Table 4:** The characteristic particle sizes of the CRFA powder.

Characteristic Particle Size (μm)			
Average Size	D_10_	D_50_	D_90_
44.9	13.7	40.9	80.2

(Note: D_x_ means that the portion of the particles with diameters smaller than D is x%.)

**Table 5:** The compacted bulk density of the coarse aggregate mixture (kg/m^3^).

The proportion of crushed aggregate 1	0%	10%	15%	20%	25%	30%
Compacted bulk density	1490	1508	1525	1532	1528	1522

**Table 6:** The compacted bulk density of the mixed aggregate (kg/m^3^).

**MFF**	**0%**	**36%**	**38%**	**40%**	**42%**	**44%**	**46%**	**48%**	**50%**
Compacted bulk density	1532	1799	1816	1835	1852	1867	1845	1840	1854

Note: MFF: the mass fraction of the fine aggregate.

**Table 7:** The mixture proportions of the concrete samples.

Design Step	No.	EF	CPI	MFF (%)	The Content of the Material (kg/m^3^)					
					Water	Cement	CRFA	Crushed Aggregate		Water Reducer
								1	2	
Initial	R11	1.05	1.00	40.0	175.1	464.1	734	220	881	7.4
9	R11	1.05	1.00	40.0	175.1	464.1	734	220	881	7.4
	R21	1.05	0.95	41.2	167.0	440.9	771	220	881	9.3
	R31	1.05	0.90	42.4	160.3	417.7	809	220	881	9.6
10	R21	1.05	0.95	41.2	168.0	440.9	771	220	881	9.3
	R22	1.05	0.95	39.2	166.7	440.9	736	228	912	8.4
	R23	1.05	0.95	37.2	166.4	440.9	699	236	943	7.5

Note: EF: excess factor; CPI: cement paste index; CRFA: completely recycled fine aggregate.

**Table 8:** The performance of the concrete samples.

Design Step	No.	Slump (mm)		Slump-Flow (mm)		Compressive Strength (MPa)		
		0 h	1 h	0 h	1 h	3 days	7 days	28 days
Initial	R11	195	205	435	375	46.5	54.1	60.0
9	R11	195	205	435	375	46.5	54.1	60.0
	R21	220	230	510	555	50.8	62.6	65.4
	R31	190	210	375	445	52.0	56.0	60.1
10	R21	220	230	510	555	50.8	62.6	65.4
	R22	220	200	490	480	54.9	60.5	64.0
	R23	220	160	560	380	61.8	62.2	71.3

**Table 9:** Material consumption for 1 m^3^ of the CRFA concrete (mix proportion R22).

No.	The Content of the Material (kg/m^3^)					
	Water	Cement	CRFA	Crushed Aggregate		Water Reducer
				1	2	
R22	154.1	407.6	680.5	210.8	843.2	7.8

**Table 10:** The material prices in Guangzhou, China, in April 2020 [33].

Material	Water	Cement	CRFA	River Sand	Crushed Aggregate 1	Crushed Aggregate 2	Water Reducer
Price (RMB Yuan/ton)	4.6	560	60	154	141	149	2400
